# Cellular, synaptic, and network effects of chemokines in the central nervous system and their implications to behavior

**DOI:** 10.1007/s43440-021-00323-2

**Published:** 2021-08-26

**Authors:** Joanna Ewa Sowa, Krzysztof Tokarski

**Affiliations:** Department of Physiology, Maj Institute of Pharmacology, Polish Academy of Sciences, 12 Smetna Street, 31-343 Krakow, Poland

**Keywords:** Chemokines, Chemokine receptors, Central nervous system, Homeostasis

## Abstract

Accumulating evidence highlights chemokines as key mediators of the bidirectional crosstalk between neurons and glial cells aimed at preserving brain functioning. The multifaceted role of these immune proteins in the CNS is mirrored by the complexity of the mechanisms underlying its biological function, including biased signaling. Neurons, only in concert with glial cells, are essential players in the modulation of brain homeostatic functions. Yet, attempts to dissect these complex multilevel mechanisms underlying coordination are still lacking. Therefore, the purpose of this review is to summarize the current knowledge about mechanisms underlying chemokine regulation of neuron–glia crosstalk linking molecular, cellular, network, and behavioral levels. Following a brief description of molecular mechanisms by which chemokines interact with their receptors and then summarizing cellular patterns of chemokine expression in the CNS, we next delve into the sequence and mechanisms of chemokine-regulated neuron–glia communication in the context of neuroprotection. We then define the interactions with other neurotransmitters, neuromodulators, and gliotransmitters. Finally, we describe their fine-tuning on the network level and the behavioral relevance of their modulation. We believe that a better understanding of the sequence and nature of events that drive neuro-glial communication holds promise for the development of new treatment strategies that could, in a context- and time-dependent manner, modulate the action of specific chemokines to promote brain repair and reduce the neurological impairment.

## Introduction

### Background

Chemokines (CHEMOtactic cytoKINES) belong to a large family of small (7–11 kDa) proteins. They were originally identified as serving chemotactic function on immune cells in late 1980 [1]. However, a decade later, the first reports revealed the prominent expression of chemokines and their receptors in the central nervous system (CNS) [2]. Since then, numerous detailed studies on the key role of these immune proteins and their receptors in the brain, also under physiological conditions, have been published, highlighting their brain-specific functions, such as the modulation of synaptic transmission [3]. It is now clear that all types of brain cells synthesize distinct chemokines and might respond to chemokine stimulation via their receptors (see Table 1). These complex cellular patterns of chemokine/chemokine receptor expression lead to intricate cell-to-cell communications. Thus, of particular interest is now the idea that CNS chemokines are not only mediators of neuroinflammation, but also they are emerging as orchestrators of neuron–glia crosstalk, which is essential in maintaining brain homeostasis [4].

**Table 1:** Summary of chemokine and chemokine receptor expression by different types of brain cells

Cell type	Chemokine/chemokine receptor
Astrocyte	CCL2, CCL2, CX3CL1, CX3CL1, CXCL10, CXCL12, CXCL16, CXCL8, CCR2*, CCR2*, CCR3, CCR4, CCR5, CCR6, CCR7*, CXCR1, CXCR2, CXCR3, CXCR4, CXCR4, CXCR4, CXCR5, CXCR6, CX3CR1*, ACKR3, ACKR3, ACKR1, CCRL2 (L-CCR)
Microglia	CX3CL1, CXCL12, CXCL14, CXCL16, CCR3, CCR4, CCR5, CCR6, CCR9, CXCR1, CXCR2, CXCR3, CXCR4, CXCR5, CXCR6, CX3CR1, ACKR3, ACKR1, CCRL2 (L-CCR)
Neural stem cells	CCR1, CCR2, CCR2, CCR3, CCR5*, CCR5*, CXCR1, CXCR3, CXCR4, CXCR4, CXCR5, CXCL14 R, CX3CR1, ACKR3
Neuron	CCL1, CCL2, CCL2, CCL2, CX3CL1, CX3CL1, CXCL12, CXCL12, CXCL12, CXCL14, CXCL16, CCR1*, CCR2*, CCR2*, CCR2*, CCR3*, CCR4, CCR5*, CCR7, CCR8, CCR9, CCR10, CXCR1, CXCR2, CXCR2, CXCR3, CXCR4, CXCR4, CXCR4, CXCR4, CXCR4, CXCR4, CXCR4, CXCR4, CXCR4, CXCR4, CXCR4, CXCR6, CX3CR1*, ACKR3, ACKR3, ACKR3, ACKR3, ACKR1
Oligodendrocyte	CCL2, CCR1, CXCR1, CXCR2, CXCR3, ACKR3

*Inconsistent results

### Chemokine and chemokine receptor classification

**Fig. 1:**
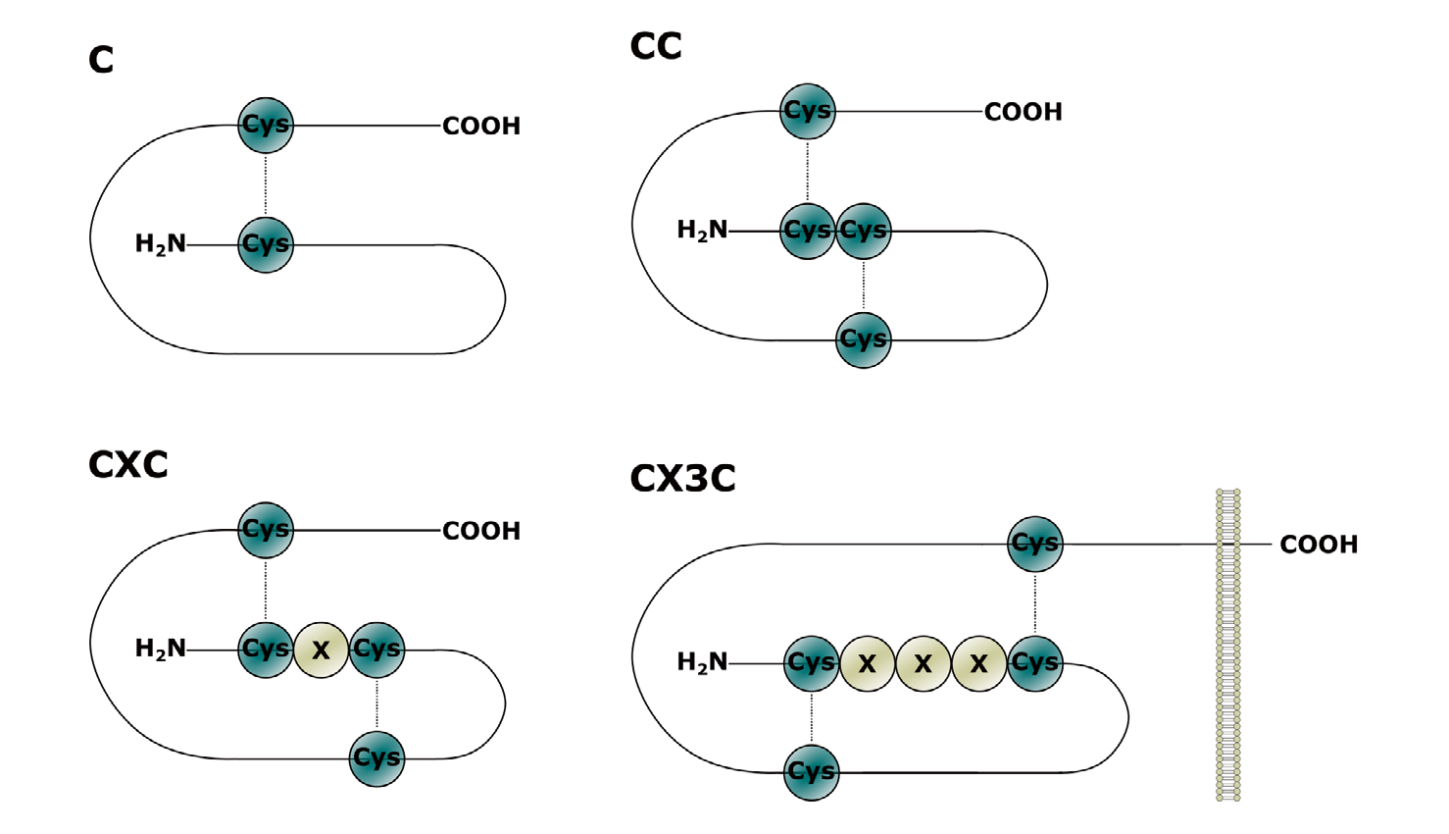
Chemokine families. Chemokines are classified into four distinct subclasses: C, CC, CXC, and CX3C according to the number and spacing of their cysteine residues in their N terminus. *Cys* cysteine residue, *X* amino acid residue, disulfide bridges are shown as dotted lines

To date, 53 human chemokines and 23 chemokine receptors have been cloned or characterized [5]. All chemokine family members share a similar tertiary structure: a flexible N terminus and N-terminal loop, followed by a three-stranded antiparallel β-sheet on which a C-terminal α-helix is folded (for reviews: [6], see Fig. 1). Although N terminus is widely acknowledged as pivotal for receptor activation, it is not sufficient in this regard, and several additional chemokine regions were subsequently identified as critical for receptor binding and signal transduction, including N-loop, or highly conserved GP (glycine–proline) motif (for review: [7]). Thus, these data illustrate that subtle structural changes in one chemokine domain may significantly alter receptor activation, resulting in unique functional outcomes for chemokine–receptor pairs.

Chemokines were originally named according to their function or from the cell type that produced them. However, a systematic nomenclature was introduced in 2000, based on the spacing of the first two cysteine residues closest to the N terminus. It consists of the subfamily designation (XC, CC, CXC, CX3C), followed by the letter “L” (denoting “ligand”), and the number according to when the gene was first isolated ([8], Fig. 1).

Chemokines exert their biological effects through cell surface chemokine receptors (CKRs), which can be divided into two families: conventional chemokine receptors belonging to the classic G-coupled protein receptors and atypical chemokine receptors. Conventional receptors are classified according to the subfamily of chemokine ligand, as mentioned above. They preferably bind and are named following the same principle as the chemokines, but replacing “L” with “R,” which denotes “receptor” (see Fig. 1). They typically transduce their signals via two major routes: G_i_ proteins and β‐arrestin [9].

Atypical CKRs, a small subset of proteins with at least four representatives [10, 11], structurally resemble conventional chemokine receptors. They bind a wide variety of chemokine ligands with high affinity and signal predominantly through β-arrestins [5].

Due to their inability to activate typical G protein-signaling pathways and thereby induce chemotactic activity, they were initially thought to scavenge chemokine ligands or function as co-receptors. However, recent data cast a new light on the complexity of the ACKRs role in regulating chemokine system signaling, also beyond inflammation (for review: [11]). Pioneer experiments demonstrated the expression of two atypical CKRs members, ACKR1 and ACKR3, in the CNS, as well as their behavioral relevance (see 2.4, Tables 1, 3).

### Chemokine signaling

The multifaceted role of chemokines in both nervous and immune systems is mirrored by the complexity of the molecular signaling mechanisms underlying their biological functions.

**Fig. 2:**
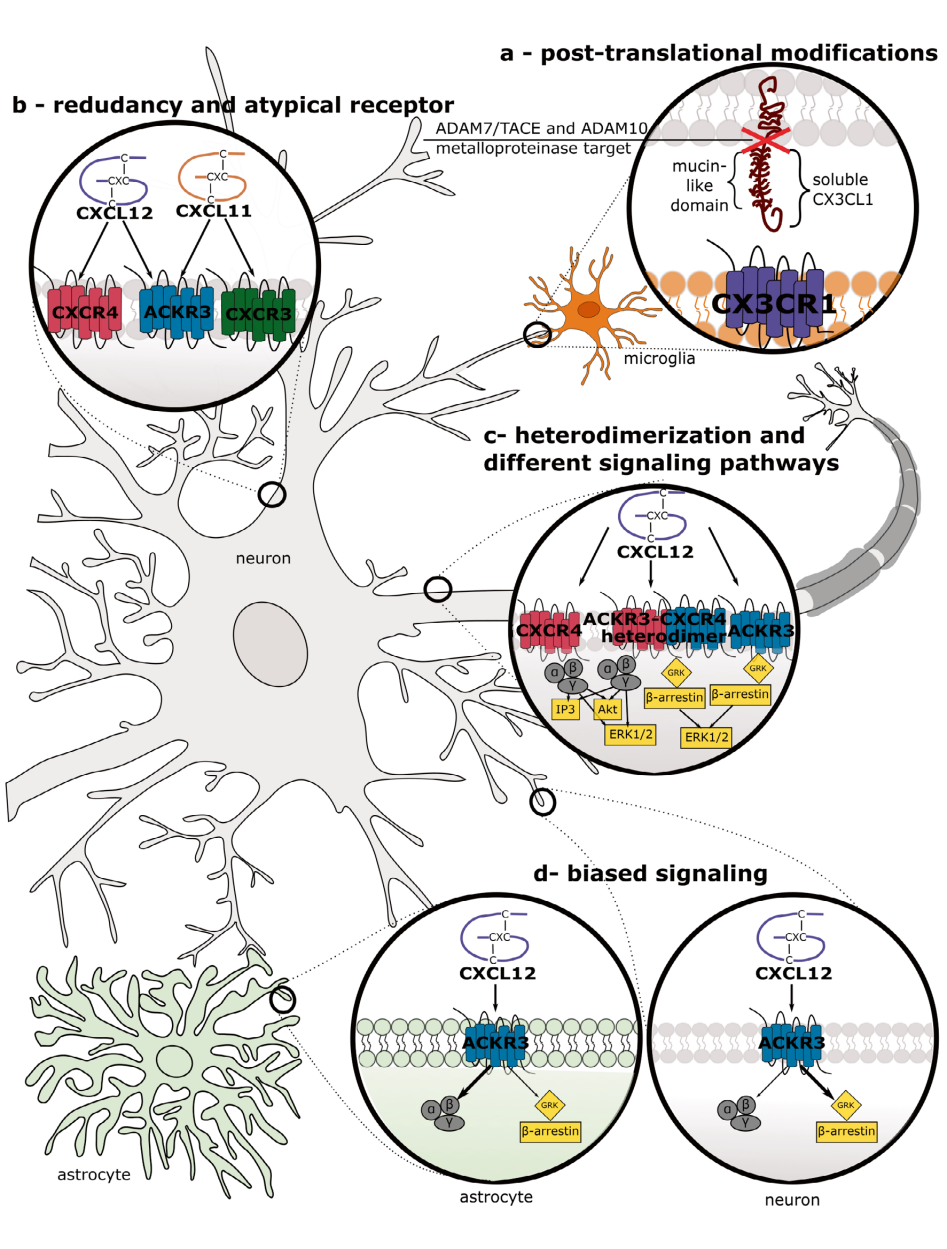
A schematic diagram provides an overview of the chemokine system’s different cellular/molecular mechanisms in the CNS. **a** Post-translational modifications exemplified by CX3CL1 transmembrane form cleavage by ADAM10 and ADAM17 proteases into its soluble variant. **b** The chemokine family redundancy is exemplified by ACKR3. It belongs to the atypical family since it was regarded as unable to induce G-coupled signaling. It binds two chemokines, CXCL11 and CXCL12. Besides the ACKR3 receptor, these two chemokines activate other chemokine receptors, namely CXCR3 and CXCR4, respectively. **c** Most chemokine receptors can form homo- and hetero-dimers. It is exemplified by the well-known CXCR4–ACKR3 complex. CXCR4 receptor is a ‘classical’ chemokine receptor, which activates G_αq/i_ signaling pathways, including PKC or (ERK) ½. As an atypical receptor, ACKR3 alone activates β-arrestin-mediated pathways, leading to receptor internalization or scavenging. However, after heterodimerization with CXCR4, it can modify ligand binding properties and receptor signaling as well as intracellular trafficking. **d** Chemokine ligand bias occurs when specific chemokines could preferentially activate different intracellular pathways, either G-protein or β-arrestin, although binding to the same receptor. It can be due to a specific ligand or receptor, as exemplified here, due to a specific cell. As suggested recently [27], when ACKR3 is activated on neurons, it signals through β-arrestin-mediated pathways, but when it is activated on astrocytes, it recruits β-arrestin-mediated pathways

Firstly, the regulation of chemokines and their receptors may be controlled by post-translational modifications (PTMs), the chemical modification of a protein after its translation, such as citrullination or cleavage by several proteases ([5], see Fig. 2A). These changes profoundly affect chemokine system activity, including chemokine activation/inactivation, the change in binding affinity, or even switching from a receptor agonist to an antagonist [5].

Another essential aspect of chemokine-induced signaling is their rich repertoire of ligand–receptor relationships (for reviews: [5, 8], see Fig. 2B). It is widely accepted that most chemokines may bind to several different receptors, whereas nearly all of CKRs may recognize various chemokine ligands. However, it becomes increasingly evident that the interactions between chemokines and CKRs are far more restricted, complex, and less redundant than previously thought, as further addressed in this section. Consequently, each chemokine/CKR pair interaction depends, for example, upon the simultaneous spatial and temporal expression of both molecules [9].

Most CKRs, like many GPCRs, constitutively exist as dimers (heterodimers/homodimers) or oligomers, thereby modifying ligand binding properties or activating different signaling pathways [12]. Heterodimers, as they are formed with other GPCRs families (e.g., with opioid receptors), underlie the synergistic or antagonistic activity of various chemokine pairs [7]. Moreover, increasing data shows that dimer formation is regulated by other factors, such as glycosaminoglycans (GAGs).

GAGs, polysaccharides present at the cell surface and within extracellular matrices, bind chemokines and thereby immobilize and accumulate them. It is an essential step for the chemokine function, as it regulates their local concentration and availability for their receptors. Considering GAGs cell-, tissue- and developmental-specific expression [13], along with their selective binding of certain chemokines, GAGs–chemokine interactions are regarded as yet another mechanism contributing to chemokines orchestration of cell-to-cell communication.

Biased signaling, also known as functional selectivity, is another feature specific for GPCR receptors ([7, 9], see Fig. 2D). Three types of biased signaling can be distinguished.

Ligand bias occurs when specific chemokines, although binding to the same receptor, could preferentially activate one of the intracellular pathways, either G-protein or β-arrestin, and their downstream elements, accordingly to cell-specific differences. Another type of bias signaling, receptor bias, occurs when a specific receptor, which typically binds multiple chemokine ligands, preferentially couples to a particular chemokine. Finally, cellular or tissue bias occurs when the same chemokine receptor pair leads to activation of distinct signaling cascades or cellular responses in different spatial contexts.

CKRs may activate different intracellular pathways in a ligand- and cell-specific context (see Fig. 2C). Chemokine receptors most often signal through canonical G protein pathways and couple either to (1) the G_αi_ subunit following inhibition of the adenylyl cyclase activity to reduce the intracellular cAMP levels or (2) G_αq_ that activates phospholipase C, leading to the formation of diacylglycerol and inositol 1,4,5-triphosphate with a subsequent increase in a protein kinase C (PKC) activity and transient elevations of cytosolic Ca^2+^ levels. However, mounting evidence describes the involvement of other intracellular signaling cascades, including distinct G protein subtypes (G_11_ or G_α12/13_), or alternative signaling targets, such as mitogen-activated protein kinase (MAPK), extracellular signal-regulated kinase (ERK) ½, Janus kinases (JAKs), and nuclear factor-κB (NF-κB) (for reviews: [14, 15]).

The mechanisms mentioned above, together with the dualistic nature of some of the chemokine ligands (i.e., a chemokine agonist at one receptor can antagonize another receptor) and the presence of non-chemokine ligands (e.g., ubiquitin, β-defensins, [5]) presents a multifaceted, highly interconnected, tightly regulated system with possible myriad functionally diverse outcomes.

## Chemokine action from synapses to behavior

The detailed mechanisms by which chemokines coordinate these composite, inter-regulated processes that enable homeostatic in the CNS, especially at the network level, remains largely unknown. One promising approach is to summarize and evaluate studies at lower levels and propagate upward to higher levels of behavior and function. Moreover, until recently, the biggest challenge was isolating the different cell-type components. Over the last decade, more advanced research strategies enable dissecting the role of glial cells in several aspects of brain functions, leading to an impressive body of literature. Therefore, this review aims to summarize the current state of knowledge of how chemokines coordinate communication between different cell types to maintain brain homeostasis by linking molecular, cellular, circuit, and ultimately behavioral levels. We hope it will provide a framework for better understanding the complex communication network between neurons and cells from their surrounding microenvironment and help identify molecular targets to counteract detrimental events and preserve brain homeostasis.

### Chemokine action at the cellular level

#### Cellular distribution of chemokine and chemokine receptors in the brain

Chemokines and CKRs are expressed by healthy neurons, microglia, astrocytes, oligodendrocytes, and endothelial cells. They are widely distributed in the CNS and involved in many physiological brain functions [16]. Table 1 shows schematically what chemokine or chemokine receptors were expressed in what type of cell. Consistently, the neuroanatomical distributions of several chemokines and their corresponding receptors overlap in many regions in the healthy CNS. They may also be co-localized within the same cell, showing a possibility of autocrine signaling. This review will focus on the five most widely studied chemokine axes (CXCL12/CXCR4/ACKR3, CX3CL1/CX3CR1, CCL3/CCL4/CCR5, CCL2/CCR2, and CXCL16/CXCR6).

Chemokines and receptors expressed in the CNS under physiological conditions are summarized in Table 1. As delineated, individual brain cells may not only co-express multiple functional chemokine receptors but also produce chemokine ligands on their own, which may influence cellular functions within the CNS, leading to complex and extensive cross-talks between different cell types.

##### CXCL12/CXCR4/ACKR3

CXCL12, along with their receptors CXCR4 and ACKR3, is constitutively expressed in the CNS, both during neurodevelopment and adulthood. These molecules are present in nearly all CNS cell types, including endothelial, glial, and notably neuronal cells (see Table 1). They are widely distributed across the CNS. Specifically, both CXCL12 and CXCR4 are co-localized with many neuropeptides, for example, in dopaminergic neurons in the substantia nigra (SN) and the ventral tegmental area [17]. Additionally, they are often co-expressed with opioid receptors in neurons and astrocytes, frequently forming heterodimers [18], which implies interactions between those two systems (see “Opioids**”**, for review: [19]). Likewise, recent evidence has begun to implicate ACKR3 in chemokine–opioid interactions, leading to behavioral manifestations (see “Chemokine action on behavior”, [20, 21]).

Moreover, ACKR3 and CXCR4 are co-expressed in the same neuronal [22] and astrocytic populations [23]. In neurons, prominent cytoplasmic expression of ACKR3 was demonstrated, whereas CXCR4 was found on the cell membrane [22], suggesting that ACKR3 may affect CXCR4 trafficking and/or coupling to other proteins [22]. Consistently, ACKR3 up-regulation was shown to enhance heterodimer formation with CXCR4, which further resulted in its internalization and degeneration [24]. As a member of the atypical chemokine receptor family, ACKR3 is thought to share some peculiar characteristics, such as the inability to induce canonical G protein signaling in response to ligand stimulation, which leads to a decrease in the extracellular chemokine concentration [25]. However, compelling evidence illustrated that application of CXCL12 results in elevated astrocytic intracellular Ca^2+^ levels following the activation of PTX-sensitive G_i/o_ proteins, leading to either proliferation or differentiation of astrocytes [23].

Although these events were previously reported to be mediated by CXCR4 (for example: [26]), a recent report has challenged this view by demonstrating that these CXCL12-dependent outcomes persist when CXCR4 receptor is blocked or depleted from astrocytes but are prevented in astrocytes with depleted or blocked ACKR3 receptor [27]. These findings suggest that ACKR3 activates classical G protein signaling pathways in these cells. It is likely that ACKR3 functions as a ligand-biased receptor in astrocytes, as CXCL11, another ACKR3 agonist [28], resulted in activation of β-arrestin2 signaling in the same experimental setting [27].

Therefore, it is tempting to speculate that depending on the cell type, ACKR3 acts either as an atypical, arrestin-coupled scavenger chemokine receptor or as a classical GPCR.

However, this issue warrants further clarification. Additionally, it was recently demonstrated that following CXCL12 treatment, ACKR3 impairs astrocytic gap-junctional communication by inducing a Cx43 internalization in a β-arrestin2-dependent manner [29], whereas CXCR4 leads to NF-Kβ activation with consequent tumor necrosis factor α (TNFα) and glutamate release from these cells [30–32]. Therefore, these findings suggest that CXCR4 and CXCR7 may serve distinct functions via different signaling pathways in astrocytes.

##### CX3CL1/CX3CR1

CX3CL1, previously known as fractalkine or neurotactin, is the only known member of the CX3C chemokine family. Together with CXCL16 (see the Section below: “CXCL12/CXCR4/ACKR3”), CX3CL1 is present in two different forms: a membrane-anchored form, which may be released as a shorter soluble form upon proteolytic cleavage by metalloproteases, such as ADAM10 or ADAM17 (for example: [33]). CX3CL1 and its receptor, CX3CR1, are widely distributed in the CNS. Besides the well-documented neuronal expression of CX3CL1, only a handful of studies revealed its presence in astrocytes, albeit at lower levels (for instance: [34]). Regarding cellular expression pattern of CX3CL1 receptor, CX3CR1, in the CNS, it remains controversial: some reports confine the localization of CX3CR1 microglia [35, 36], whereas several lines of biochemical, immunohistochemical, and electrophysiological evidence documented the presence of the functional CX3CR1 receptor also in hippocampal, hypothalamic, cerebral, striatal, and dorsal raphe nucleus (DRN) neurons.

In the CX3CR1-GFP knock-in mouse line, the CX3CR1 receptor gene has been replaced by a green fluorescent protein (GFP) reporter [37]. Based on CX3CL1 and CX3CR1 expression, this genetic strain provides the most studied model to investigate the consequences of neuron–microglia communication following CX3CL1–CX3CR1 axis dysregulation.

##### CXCL16/CXCR6

As mentioned above, CXCL16 is another transmembrane chemokine that, upon cleavage by metalloproteases, may be secreted as a soluble form. Although its role was initially identified as related to neuroinflammation, evidence documenting that CXCL16 exerts a role in physiological processes has just begun to emerge [38–40]. Considering expression in the CNS, CXCL16, together with its unique receptor CXCR6, is constitutively expressed in distinct CNS cells (see Table 1), further highlighting their involvement in mediating cell-to-cell communication or synaptic transmission (see also: 2.2.1, [40]). Their effects in the CNS are still largely unexplored; however, recently, CXCL16/CXCR6 expression changes are becoming associated with prenatal alcohol consumption [41] or mild traumatic brain injury [42].

##### CCL3/CCL4/CCL5/CCR5

Unlike CX3CR1, CCR5 has many chemokine ligands, including CCL3, CCL4, and CCL5 [43, 44]. In the CNS, CCR5 is highly present in microglia, whereas astrocytes, oligodendrocytes, and neurons express this receptor to a lesser extent. Finally, CCR5 has emerged as a modulatory element involved in synaptic functions (see “Chemokines action at synaptic/axonal/dendritic level”) and behavioral manifestations, including learning and memory impairments (see Sect “Learning and memory”).

##### CCL2/CCR2

CCL2/CCR2 are constitutively expressed in neurons and glia in several different regions of the CNS. Similar to CXCL12, CCL2 was also shown to co-localize with classical neurotransmitters, as discussed below. In the spinal cord, constitutive CCL2 expression has been detected in DRG neurons. Considering non-neuronal cells, astrocytes and microglia are the main sources of CCL2 production in the brain. However, using CCR2-RFP knock-in mice, one study documented no expression of CCR2 in the CNS under physiological conditions, regardless of cell type [45]. Therefore, further investigations are warranted to clarify these contradictory results.

#### Neuro-glial cross-talk

Having a diverse cellular distribution in the CNS, chemokines and their receptors plays a key role as mediators of homeostatic crosstalk between neurons and glia. Efficient communication between cells is a key element in establishing and maintaining a healthy microenvironment essential for proper brain functioning. Figure 4 presents many different mechanisms regulating such communication, exemplified by CX3CL1, as their actions are the most studied. Moreover, depending on the context, chemokines or glial cells can trigger different mechanisms of neuroprotection or neurotoxicity (for example: [46]). Glia are emerging as highly dynamics and heterogeneous cells, which constantly monitor brain parenchyma to sense local perturbation, and they express a rich repertoire of chemokine receptors. Thus, it can be speculated that these communicating molecules might orchestrate neuron–microglia–astrocyte–oligodendrocytic actions, linking the influence of the environment to brain function and behavior. Therefore, dissecting mechanisms of neuron–glial communications in the brain is of high importance, as identifying molecular targets may be used to counteract brain damage and preserve brain homeostasis.

This section will briefly introduce glial functions underlying their capacity to maintain homeostasis and an example of a chemokine-modulated cellular circuit.

##### Astrocytes

Since the tripartite synapse concept emerged in 1999 [47], it is well established that astrocytes are involved in the dynamic regulation of synaptic transmission (for example: [48]). To react to changing microenvironment, they are endowed with a great variety of voltage and ligand-operated ion channels [49]. Astrocytes display a wide variety of Ca^2+^ signals, particularly relevant in astrocytic signaling, as Ca^2+^ fluctuations are thought to integrate environmental information and generate functional outputs, such as releasing gliotransmitters, including glutamate, D-serine, GABA, ATP, adenosine, or TNFα [50].

Astrocytes are enriched in gap junctions, by which they form broad cellular networks comprised of hundreds of cells, allowing for the intercellular diffusion of ions, second messengers, cyclic AMP, Ca^2+^, glutamate, ATP, among others. Gap junctions are formed by connexins, mainly by Cx43 and Cx30, which differ in their expression. Despite their scale, such networks were illustrated to preferentially couple one astrocyte over the other. Therefore neighboring astrocytes may not necessarily be connected by gap junctions, and such preference may be of functional relevance. Moreover, gap junction networks were demonstrated to play an essential role in complex brain functions, including cognition and behavior (for a review: [50]). As ACKR3 was demonstrated to induce the internalization of Cx43 [29], future studies aimed at establishing its functional significance and whether other chemokine receptors may elicit similar actions are warranted.

##### Microglia

Similar to astrocytes, microglia were originally regarded as “guardians of immune reaction,” protecting the CNS from pathogens. CX3CL1–CX3CR1 signaling, a widely used model for investigating neuron–microglia communication (see “Microglia”), is implicated in a broad spectrum of microglial physiological properties, such as dynamic surveillance of the brain 3parenchyma and synaptic pruning. In turn, these functions may alter synaptic plasticity and behavior (see Sects: “Hebbian plasticity—LTP and LTD” and “Learning and memory”, see Fig. 4). Moreover, a recent study demonstrated that the depletion of CX3CR1 might affect the ratio of microglial subpopulations (i.e., MCHII^+^ and CD206^+^), which positively correlated with morphological (microglial deramification) and behavioral changes (i.e., social submissiveness) changes (see “Emotional behavior”, [51]).

##### Oligodendrocytes

Oligodendrocytes (OLs) are a type of glial cell that recently has attracted much attention due to their novel functions in synaptic activity. OLs were known for their ability to form myelin wrapping around neuronal axons. However, recent studies demonstrated that clemastine, a drug associated with remyelination, affected neurotransmission [52, 53] and behavior [54–56]. Moreover, as shown by optogenetic studies, neuronal activity was reported to boost myelination, in which stimulation of neuronal activity increased the proliferation and differentiation of oligodendrocyte progenitor cells (OPCs), leading to increased axonal myelination [57, 58]. Considering that stimulated axons had an increased chance of being myelinated compared to neighboring non-stimulated axons [59], these observed changes might lead to experience-driven modulation of neural circuitry.

OLs are described to crosstalk with astrocytes by coupling proper connexins and forming gap junctions [60] and by chemokine–chemokine receptor interactions, for example, via CCL2/CCR2 axis [61]. Moreover, ACKR3 was reported to be upregulated in OPCs in the subventricular zone (SVZ) in the model of multiple sclerosis (MS), suggesting its remyelination potential [62].

Thus, OLs are emerging as plastic and dynamic players in relevant brain functions; however, more studies are needed. Moreover, despite chemokine involvement in MS and a vast repertoire of chemokine receptors on OLs, functional consequences of chemokine receptors activation on OLs function and the synaptic transmission, neuronal networks, and behavior are largely unknown.

To sum up, over the past decade, knowledge of the role of glial cells in the CNS has changed dramatically. Considering high heterogeneity not only among neurons but also astrocytes, microglia, and oligodendrocytes [63–65], along with the assumption that each subpopulation may reveal unique properties, reflecting different functions, further studies investigating this issue are of high importance.

##### Cellular consequences of CXCL12/CX3CL1/CXCL16/CCL2 action on neuron–astrocyte–microglia communication

Here, we will summarize a simplified model of coordinated actions of neuron–glia interactions fine-tuned by chosen chemokines and gliotransmitters.

**Fig. 3:**
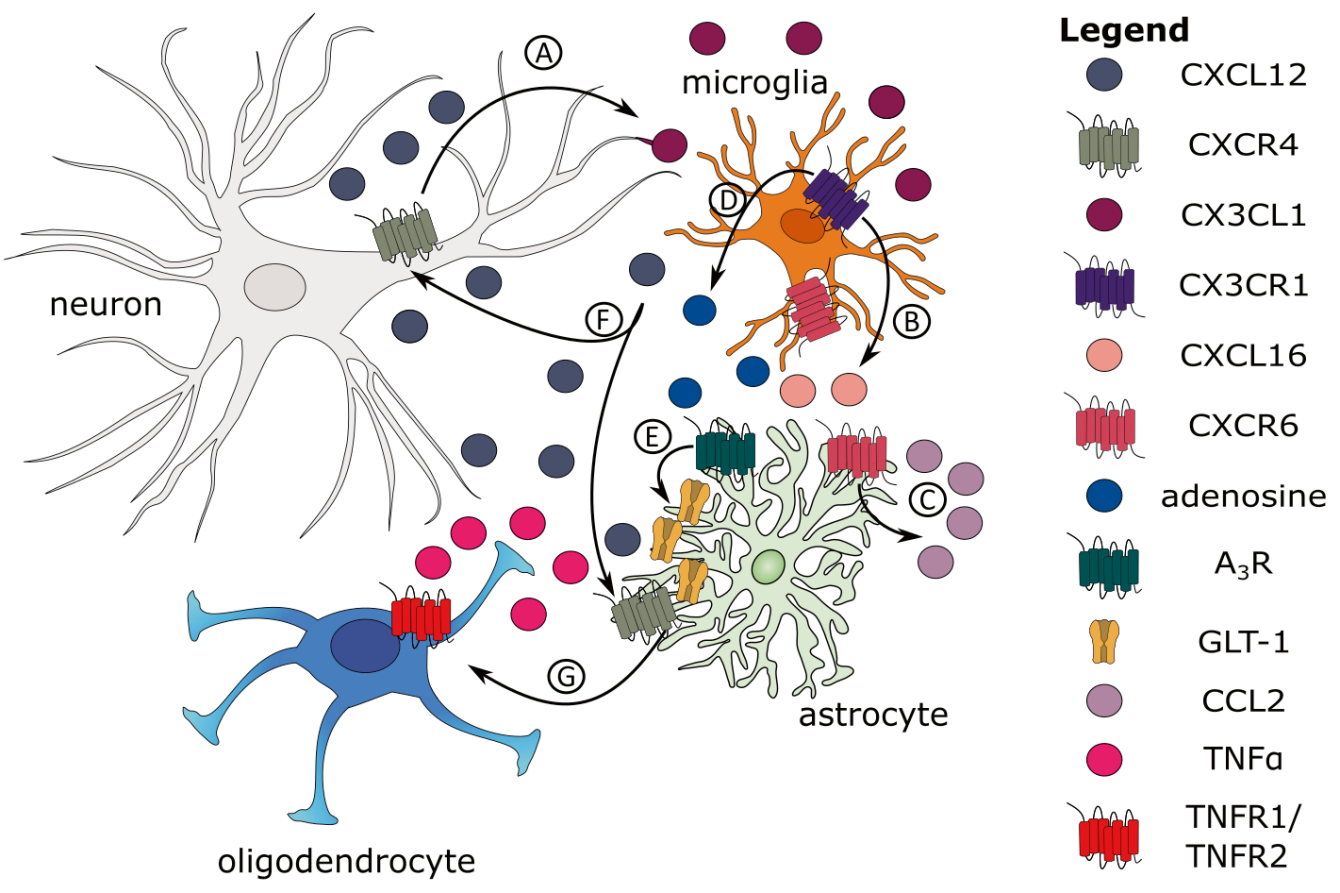
Cartoon summarizing a simplified model of coordinated actions of neuron–glia interactions fine-tuned by chosen chemokines and gliotransmitters. **a** CXCL12 acts on neuronal ADAM17 [68] and releases soluble CX3CL1. **b** Upon microglial CX3CR1 activation, CXCL16 is secreted from microglia ([38]), and **c** acts on astrocytes by inducing the additional release of CCL2. Notably, other soluble factors that mediate CXCL16-dependent neuroprotection cannot be excluded since blocking CCL2 activity dramatically reduces, but did not fully abolish, its ability to preserve neurons ([38]). **d** CX3CL1 acts on microglia and releases the adenosine that exerted their effects on astrocytes by binding to A_1_R, and **e** consequently induces the up-regulation of the astrocytic GLT-1 transporter ([78]). **f** CXCL12 is produced by microglia and acts, for example, on CXCR4 on neurons and astrocytes [30]. **g** TNFα leads to remyelination [79]

As mentioned above, CX3CL1 is the most widely studied chemokines in relation to neuron–microglia crosstalk and regulation of microglial activity. Data illustrate that many environmental triggers can up-regulate CX3CL1 expression, including an insult [66], learning [67], or the presence of another chemokine, CXCL12, via its action on neuronal ADAM17 [68] (Fig. 3A). Upon CX3CR1 activation, CXCL16 was reported to be secreted from astrocytes and microglia (see Fig. 3B, [38]). CXCL16, consequently, acts on astrocytes by inducing the additional release of CCL2, with a mechanism that requires adenosine A3 Receptor subtype (A_3_R) activity (see Fig. 3C, [38, 39, 69]). Notably, other soluble factors that mediate CXCL16-dependent neuroprotection cannot be excluded since blocking CCL2 activity dramatically reduces, but did not entirely abolish, its ability to preserve neurons (Fig. 3C, [38]). Therefore, mechanisms underlying the neuroprotective activity of CX3CL1-induced CXCL16 require the neuronal-astrocytic-microglial interplay and the activity of the A3R, with consequent release of CCL2 by glial cells.

CX3CL1 was shown to exert neuroprotective actions against Glu-induced excitotoxicity, with mechanisms fully dependent on A_1_R [70, 71] and at least partially dependent (via CXCL16) on A_3_R [38, 39]. In contrast, NMDA-induced toxicity, mainly mediated by NR2B-containing extrasynaptic NMDARs, was reported to require A_2A_R and the presence of d-serine [72, 73]. These results highlight that modulation of glial function by adenosine is essential in the context of neuroprotection, as both microglia and astrocytes release it as a gliotransmitter.

In addition to being secreted by astrocytic activation by CXCL16, CCL2 was shown to be released by these cells upon CCL5 [74], TNFα [75], or norepinephrine [76] stimulation, illustrating that astrocyte-derived CCL2 mediates the neuroprotective effect of many different excitotoxic events. Additionally, it is well-established that astrocytes might also exert a neuroprotective role upon brain insult by buffering extracellular Glu [77]. Evidence illustrated that CX3CL1, acting on microglia, induced the production and release of adenosine that exerted their effects on astrocytes by binding to A_1_R (Fig. 3D), and consequently inducing the up-regulation of the astrocytic GLT-1 transporter (Fig. 3E, [78]). Microglia can also release CXCL12, which then acts, for example, via astrocytic CXCR4 and, in turn, produces TNFα, leading to remyelination (see Fig. 3F,G, [30, 79]).

To sum up, the mentioned above findings strongly support the concept that, in the CNS, chemokines are key communicating molecules between neurons and glia cells aimed at preserving brain functions. Please note that this is a simplification of neuron-microglia-astrocyte cross-talk in the context of neuroprotection, and many other factors and connections are omitted for the sake of clarity.

### Chemokine action at synaptic/axonal/dendritic level

Accumulating data reveals that chemokines modulate neuronal electrical activity through both postsynaptic (i.e., including electrophysiological output following chemokine receptor activation, such as Ca^2+^ transients, intrinsic membrane properties by activation/inactivation of particular channels/receptors) and presynaptic (i.e., including presence in synaptic vesicle and (co-)release of neuropeptides/neurotransmitters/gliotransmitters) mechanisms ([3]). In addition, mounting evidence shows interactions between the chemokine system and “classical” neurotransmitters or neuromodulators. Considering complexity resulting from differences in postsynaptic cell machinery, including (1) diverse signaling pathways, (2) a rich repertoire of channels and receptors present among cells expressing a particular chemokine receptor and channels, (3) dual/opposite action of the same chemokine, (4) multiple chemokine ligands for particular chemokine receptors or few receptors binding one particular chemokine ligand present at the same cell, as well as possible (5) indirect effects of glial components, chemokines may exert mixed, both pre- and postsynaptic (for example: [80]) and/or functional outcomes due to chemokine receptor activation may differ in a cell- and circuit-specific manner (for example: [81]). Therefore, this chapter aims to summarize electrophysiological studies, focusing on chemokines’ interaction with other systems and glial cells (see Table 2). Additionally, based on the premise that dendritic spines are a structural manifestation of synapses, we will briefly outline the evidence that chemokines also regulate these membrane protrusions.

**Table 2:** Summary of chemokines action in electrophysiological studies

Chemokine	Concentration (nM)	Preparation	Structure/cells	Animal	Age	Sex	Glia involvement	Effect	Reference
Patch-clamp									
CCL2	1	Acute slices	RN, Serotonergic neurons	Mice	0.5–1 month	F	?	Hyperpolarization, ↓ excitability, ↓ membrane potential	[114]
CCL2	120	Acute slices	Spinal cord, Lamina II neurons	Mice	1 month (3–4 weeks)	M	?	↑ Amplitude and frequency of sEPSC, ↑ in AMPA- and NMDA-induced current	[92]
CCL2	120	Acute slices	Hippocampus (CA1, CA3, DG), primary somatosensory cortex, pyramidal neurons or granule cells	Mice	0.5 month	Both	?	CCR2-dependent ↑ mEPSC frequency, but not amplitude, ↑ The amplitude of eEPSC, ↑ AMPA/NMDA ratio, ↑ Excitability, no difference in PPR or mIPSC	[90]
CCL2	3–50	Primary neuronal cultures	Spinal cord	Rat	E14–E15	Both	?	Inhibition of GABA-induced currents, A no effect on membrane properties	[102]
CCL2	10	Acute slices	SN, dopaminergic neurons	Rat	2 months	M	?	↑ Membrane resistance (closing K^+^ channels), ↑ Excitability of DA neurons	[106]
CCL2	100	Acutely dissociated cells or acute slices	DRG	Rat	1 month	F	None	Depolarization (higher % of cells in neuropathic pain model), ↓ Rheobase, ↑ AP width, Activation of a non-voltage-dependent depolarizing current with characteristics similar to a nonselective cation conductance, inhibition of a voltage-dependent outward current	[169]
CCL2	13–100	Primary neuronal cultures	Cerebellum, Purkinje neuron	Rat	E20	Both	?	↓ Excitability	[177]
CCL2	2.3	Acute slices	Hippocampus (CA1), pyramidal neurons	Rat	0.5–1 month	M	?	CCR2-dependent-depolarization and ↑ Excitability, ↑ In frequency of sEPSC,	[89]
CCL2	24,122.5	Acute slices	Hippocampus, microglia	human (patients with temporal lobe epilepsy)	?	?	Microglial recordings	↑ Ca^2+^-dependent K^+^ channel currents	[180]
CCL21	10,000	Primary microglial cultures	Cerebellum, microglia	Mouse	?	Both	Microglial recordings	CXCR3-dependent ↑ in Cl^−^ channel current	[178]
CCL22	50	Neuronal and HEK cultures	HEK293 cells stably expressing N-type calcium channels (G1A1 cells), DRG/NTS neurons	Rat	2–5 days	Both	?	Inhibition of N-type calcium channels in HEK cells and DRG neurons	[179]
CCL3	100	Primary neuronal cultures	Cerebellum, Purkinje neuron	Rat	E16-17	Both	?	↑ Excitability (lower threshold)	[174]
CCL5	10	Primary neuronal cultures	Hippocampus	Rat	E17-18	Both	?	↓ Frequency of sEPSC and Ca^2+^ currents	[2]
CCL5	50	Neuronal and HEK cultures	HEK293 cells stably expressing N-type calcium channels (G1A1 cells), DRG/ NTS neurons	Rat	2–5 days	Both	?	Inhibition of N-type calcium channels in HEK cells and NTS neurons	[179]
CX3CL1	100	Primary neuronal cultures	Hippocampus	Rat	E17-18	Both	?	↓ Frequency of sEPSC and Ca^2+^ currents	[2]
CX3CL1	1–100	Neuronal and HEK cultures	HEK293 cells stably expressing N-type calcium channels (G1A1 cells); DRG neurons; NTS neurons	Rat	2–5 days	Both	?	Inhibition of N-type calcium channels in HEK cells and DRG neurons	[179]
CX3CL1	5	Neuronal hippocampal cultures	Hippocampus	Mice, CX3CR1^−/−^	1–2 days	Both	Using medium from microglial cell line/adding CX3CL1 in the medium	↓ AMPA-current,	[70]
CX3CL1	100	Acute slices	Hippocampus (CA1), pyramidal cells	Rat and mice (CX3CR1^−/−^)	0.5–1 month	Both	?	CX3CR1-dependent ↓ eEPSC and sEPSC the amplitude	[82]
CX3CL1	5	Neuronal cultures	Hippocampus	Mice, C57BL/6 or A_1_R^−/−^, A_2A_R^−/−^, A_3_R^−/−^	1–2 days	Both	?	CX3CL1 ↓ amplitude of eEPSC; in the presence of adenosine enzyme—the effects were more robust (tonic effect of adenosine even in the absence of CX3CL1)	[83]
CXCL12	25	Acute slices	Hypothalamus, AVP neurons	Rat	0.5–1 month	M	?	CXCR4-dependent ↓/↑ in excitability	[111]
CXCL12	0.1–10	Acute slices	Hypothalamus, MCH neurons	Rat	0.5–1 month	M	Not dependent on Glu secretion from astrocyte (presence of mGluR antagonist MCPG)	CXCR4-dependent GIRK-current induced hyperpolarization, ↓ excitability and membrane resistance	[80]
CXCL12	10	Primary neuronal cultures or human SH-SY5Y cells	Hypothalamus	Mice	E15	Both	Not dependent on Glu secretion from astrocyte (presence of mGluR antagonist MCPG)	↓ Excitability, ↓ sodium inward currents, ↓ Delayed rectifier potassium currents	[109]
CXCL12	10	Acute slices	SN, dopaminergic neurons	Rat	0.5 month	M	Glutamatergic inward current resistant to TTX and not mediated by CXCR4—probably non-neuronal	CXCR4-dependent ↑ sIPSC frequency and amplitude, ↑ mIPSC frequency	[17]
CXCL12	0.1–10	Acute slices	SN, dopaminergic neurons	Rat	0.5 month	M	?	CXCR4-dependent increase in the amplitude of N-type Ca + currents	[105]
CXCL12	1–10	acute slices	DG, immature granular neurons	Rat	< 0.5 month	M	?	↑ Excitability, ↓ Latency of the AP (32% of cells)	[170]
CXCL12	50	Primary neuronal cultures	Hippocampus	Rat	E16-17	Both	?	↓ Frequency of sEPSC and Ca^2+^ currents	[2]
CXCL12	50	Neuronal and HEK cultures	HEK293 cells stably expressing N-type calcium channels (G1A1 cells); DRG neurons; NTS neurons	Rat	2–5 days	Both	?	Inhibition of N-type calcium channels in HEK cells	[179]
CXCL12	10	Acute slices	SN, dopaminergic neurons	Rat	0.5–1 month	M	?	CXCR4-dependent ↑ firing frequency, ↑bursting firing pattern	[107]
CXCL12	25	Acute slices	Cerebellum, Purkinje cells	Rat	0.5–1.5 months	M	?	↓ eEPSC amplitude, ↓ NMDAR	[95]
CXCL12	25	Acute slices	Cerebellum, Purkinje cells	Rat	1–1.5 mounts	M	Discussion about not fully blocked inward current that may reflect extrasynaptic glutamate, possibly released from the surrounding glia	Slow inward current, ↑ spontaneous synaptic activity, ↑ frequency of synaptic currents (mainly GABAergic)	[99]
CXCL16	10	Acute slices	Hippocampus	Mice (A_3_R^−/−^)	1 month	Both	Mediation of microglia—recordings in minocycline	↑ mIPSC frequency, ↑ PPR of eIPSC, and ↑ mEPSC frequency	[40]
CXCL8	10	Acutely dissociated neurons	Septum	Rat	0.5 month	Both	?	Closure of L- and N-type channels	[172]
Extracellular recordings									
CCL2	10 × higher concentration than in control mice	Acute slices	Hippocampus (CA1)	Mice with CCL2 overexpression in astrocytes	7–9 months	Both	Expression of CCL2 specifically in astrocytes	↓ Synaptic transmission, ↓ Neuronal excitability, ↑ short-term synaptic plasticity	[93]
CCL2	422.4	Acute slices	Hippocampus(ca1,ca3,dg), primary somatosensory cortex	Mouse	2–3 months	M	?	↓ fEPSC and LTP, no changes in fNMDA and LTD	[87]
CX3CL1	x	Acute slices	Hippocampus	Mice, CX3CR1^−/−^	3	M	?	Impaired LTP in KO	[143]
CX3CL1	x	Acute slices	Hippocampus	Mice, with CX3CR1^−/−^ and A_3_R^−/−^	0.5 or 1.5	M	?	↑ LTD at P15, but no differences at P40	[118]
CX3CL1	2	Acute slices	Striatum	Mice (wild type and R6/1 mice)	4 months	M	Blocking microglia activity with minocycline	CX3CL1 restores LTD in R6/1 mice	[183]
CX3CL1	2	Acute slices	Hippocampus	Mice, with CX3CR1^−/−^ and A_3_R^−/−^	1.5–2 months	both	Based on their previous finding that CX3CL1 induce adenosine in microglial cultures	CX3CR1- and A_3_R-dependent inhibition of the LTP induction	[145]
CX3CL1	x	Acute slices	Hippocampus	Mice, with CX3CR1^−/−^ and A_3_R^−/−^	3 months	F	?	No change due to environmental enrichment LTP	[144]
CX3CL1	5	Acute slices	Hippocampus (CA1)	Mice, C57BL/6 or CX3CR1^−/−^, A_1_R^−/−^, A_2A_R^−/−^, A_3_R^−/−^	1 month	M	Blocking microglia activity with minocycline	Microglia-driven A_2A_R-dependent and D-serine-dependent transient ↑ NMDAR function, not altered PPF	[72]
CX3CL1	0.2–20	Acute slices	Hippocampus (CA1)	Mice, C57BL/6 or CX3CR1^−/−^, A_1_R^−/−^, A_2A_R^−/−^, A_3_R^−/−^	0.5–1 month	Both	?	↓ fEPSP, suggesting that CX3CL1-induced depression shares at least some molecular mechanisms in common with LTD	[175]
CX3CL1	0.5	Acute slices	DG	Rat	2.5 month	M	A separate set of experiments (not electrophysiological) on glial and organotypic cultures	↓ fEPSC and completely blocked LTP, ↑ LTP in the presence of blocked GABA_A_	[67]

?—no data

x—no application of chemokine, but the comparison between genotypes/groups

#### Glutamate

**Fig. 4:**
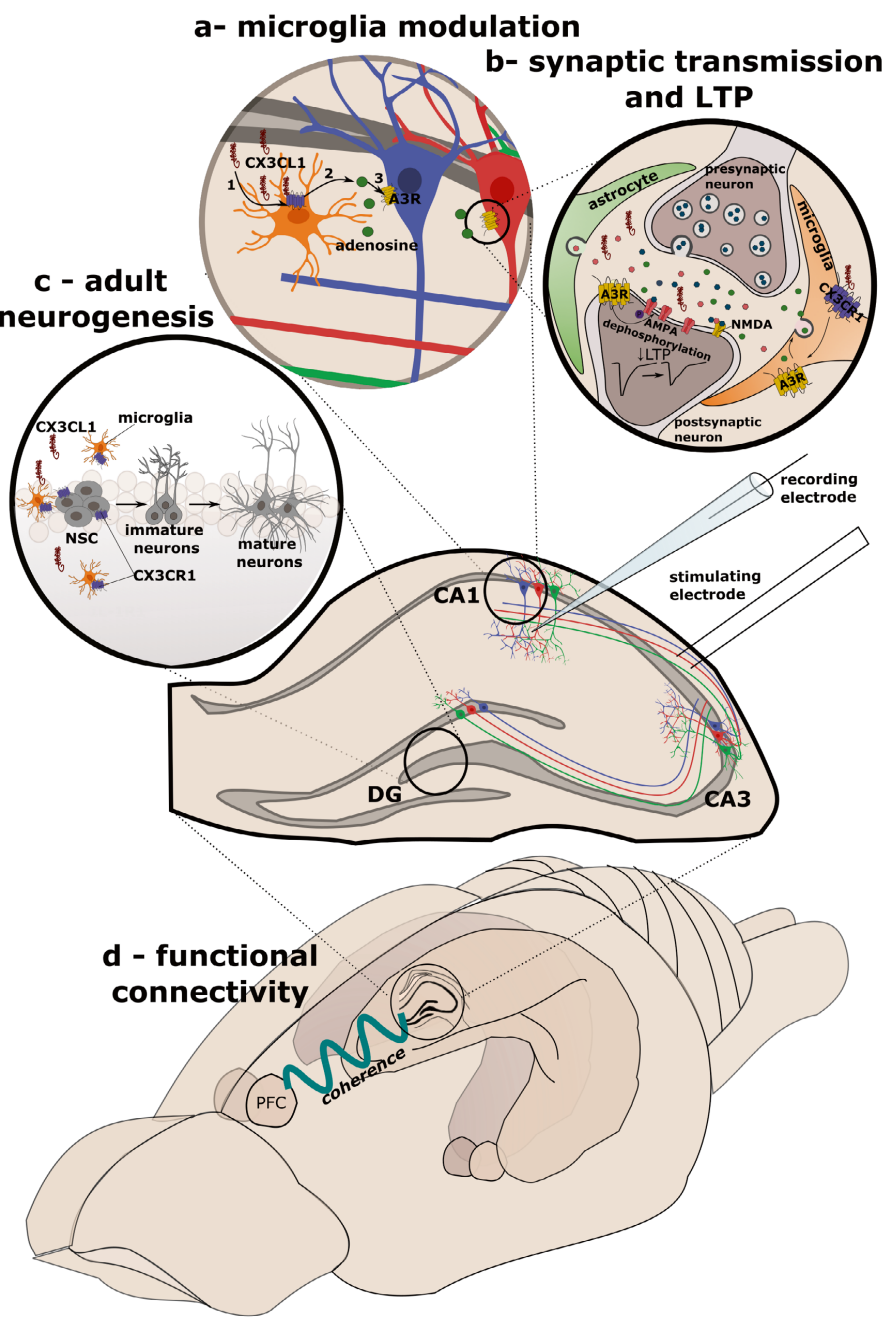
A simplified schematic diagram that provides an overview of the chemokine system’s synaptic/network mechanisms in the CNS exemplified by the CX3CL1/CX3CR1 axis. **a**, **b** In CA1, CX3CL1 reduces glutamatergic synaptic transmission and amplitude of LTP by indirect, microglia action. Briefly, CX3CL1 activates CX3CR1 on microglia, and thus, these cells secrete adenosine. Then, adenosine, via A_3_R receptors on postsynaptic neurons, dephosphorylates the AMPA subunit, thus leading to reduced glutamatergic transmission and reduction in the LTP. Based on **c**, chemokines regulate adult neurogenesis. Specifically, CX3CL1 may play a role in hippocampal neurogenesis by inhibiting the release of IL-1β from microglial cell types. **d** CX3CL1 is also documented to modulate coherence of hippocampal–prefrontal cortex connection. Schematic based on results of [120]

In the hippocampus, a region crucial for cognition, CX3CL1 was noticed to reduce the amplitude of both synaptic [82] and AMPA-evoked currents [70, 82], which suggest a postsynaptic mechanism and probably is a result of PKA-related phosphorylation of GluR1 Ser845 ([82], Table 2). However, further investigation of this effect revealed that CX3CL1 did not act directly on postsynaptic neurons, but it stimulated microglia to secrete adenosine, which in turn inhibits glutamatergic transmission by its receptors, adenosine A3 receptor (A_3_R), and to the lesser extend adenosine A1 receptor (A_1_R) ([70, 71, 83], see Fig. 4B).

Adenosine is an endogenous modulator of brain functions with increasingly evident actions on synaptic transmission [84] or glia [85], which acts by binding to its GPCR presynaptic receptor subtypes (A_1_, A_2A_, A_2C_, A_3_) and alter neurotransmitter release probability [86]. Further evidence indicates that the activation of A_1_R and A_3_R results in a decrease in synaptic transmission, whereas A_2A_R exerts the opposite effect, manifested as CX3CL1-induced increase of evoked EPSC (eEPSC) in the blockade of A_1_R and A_3_R receptors [83].

CX3CL1 was also shown to trigger D-serine, NMDAR co-agonist released from glial cells, and potentiate NMDAR function in an A_2A_- and D-serine-dependent manner, which was driven by microglial cells [72]. Therefore, the CX3CL1-induced adenosine release may affect glutamatergic neurotransmission by a mechanism involving a combination of three different AR subtypes and both glutamatergic receptors. Nevertheless, the opposite effects of CX3CL1 on AMPAR and NMDAR functions are nicely correlated with the difference between A_3_R/A_1_R and A_2A_R receptors. Moreover, the depressed glutamatergic transmission following CX3CL1 and adenosine application corresponds with results from studies of synaptic plasticity (see section Hebbian plasticity—LTP and LTD).

Another chemokine, CCL3, with its receptor, CCR5, elicited reduced glutamate transmission by a postsynaptic mechanism in the hippocampus [87]. It should be noted that the potential involvement of glial cells was not investigated in this study despite well-documented astrocytic secretion of CCL5 and CCL3, ligands of CCR5 (for review: [88]). Thus, further experiments identifying the properties and functions of each of neuronal, microglial, astrocytic and, oligodendrocytic compartments remain to be addressed.

In contrast to CX3CL1, other chemokines were reported to promote excitatory synaptic transmission in the hippocampus. Specifically, (1) CXCL16 was shown to increase excitatory synaptic transmission by an A_3_R-mediated presynaptic mechanism and microglial modulation [40], and (2) treatment with CCL2 resulted in an increase in the frequency of spontaneous and miniature excitatory synaptic currents (sEPSCs/mEPSCs), and consequently, membrane depolarization and increased excitability [89, 90]. As CCL2 did not affect the intrinsic membrane properties of pyramidal neurons, such as the input resistance, the more excitable state of these cells is probably due to an increased concentration of glutamate in synapses [90]. In addition, CCL2 application onto hippocampal CA3 pyramidal neurons, DG granule cells, layer 2/3 pyramidal neurons of the primary somatosensory cortex [90], cerebral Purkinje cells [91], or spinal cord lamina II neurons [92] produced similar electrophysiological responses, i.e., the potentiation of AMPA and/or NMDA synaptic currents or AMPA/NMDA ratio, indicating a postsynaptic effect through unsilencing of ‘‘silent’’ synapses [90].

However, as adenosine was suggested to be a downstream element of both CX3CL1 and CXCL16 signaling, along with the facts that CXCL16 was shown to act directly on astrocytes leading to a secretion of CCL2 [38], and microglia, together with astrocytes, were identified as important cellular sources of CCL2, glial modulation of the observed effect is very plausible. By employing transgenic mice with chronically elevated expression of CCL2, specifically in astrocytes, it was shown that CCL2 reduced synaptic transmission and neuronal excitability, whereas it enhanced short-term synaptic plasticity [93]. However, although both acute and chronic CCL2 leads to presynaptic alternations in glutamatergic input to the CA1 area of the hippocampus, the molecular mechanism underlying this discrepancy remains unclear. As those experiments varied regarding: (1) CCL2 application (acute vs. chronic), (2) source (astrocytic vs. bath-applied during recordings), (3) method (patch-clamp vs. extracellular recordings, see Table 2), more studies clarifying this issue are needed.

In relation to astrocytes, it is well established that these cells actively participate in synaptic transmission. For instance, considering the high expression of CXCR4 in astrocytes, pioneering studies have shown that CXCR4 activation triggers a burst of exocytosis, leading to the release of Ca^2+^ from internal stores [31]. These results further support the notion that astrocytes can respond to external stimuli and communicate via fast release of glutamate on a millisecond time scale [32].

In addition to glutamate, CXCR4 is also known to release prostaglandins and TNFα upon activation by CXCL12 on astrocytes [30], which has critical functional consequences on the neuronal networks as described in “Synaptic scaling”. Evidence indicates that CXCR4 can also affect NMDAR. For instance, CXCR4 receptors were shown to be presynaptically located in hippocampal noradrenergic and glutamatergic nerve endings. They were connected with presynaptic NMDA receptors, suggesting that CXCR4–NMDAR interactions can regulate both noradrenaline and glutamate exocytosis ([94]). Moreover, in the cerebellum, CXCR4 activation results in a decrease in NMDAR function, and thus, leads to synaptic depression ([95], see Table 2), probably by CXCL12-induced reduction in NR2B expression subunit of NMDAR [96]. CXCR4 is also widely expressed on microglia, and thus being able to react to astrocytic glutamate.

Moreover, recent studies have begun to elucidate that connexins, the gap junction forming proteins highly expressed on astrocytes, scale synaptic transmission, and plasticity. For instance, while Cx43, the most abundant astrocytic connexin, does not appear to regulate astrocytic membrane properties, a growing body of evidence suggests that it forms hemichannels and regulates the excitatory synaptic transmission of hippocampal CA1 pyramidal cells via ATP signaling [97]. Moreover, further experiments demonstrated that Cx43 reduced the amplitude of NMDA-mediated EPSCs in the prefrontal cortex. This effect could be rescued with the presence of D-serine in the extracellular medium [98]. It is worth noting that recently ACKR3, an atypical receptor of CXC12, was suggested to internalize Cx43 [21]. Therefore, these findings support the notion that ACKR3-induced Cx43 internalization in the hippocampus or prefrontal cortex leads to dysfunction in synaptic transmission and, in turn, functional consequences on learning and memory. However, to our knowledge, there are no data that would confirm this hypothesis.

The importance of oligodendrocytes in the regulation of synaptic transmission is just now being appreciated, and only scarce evidence has been reported so far. However, few studies suggested two molecular targets, which may be involved in this phenomenon: BDNF and glutamine synthetase (GS). Recently, it was shown that oligodendrocyte-derived BDNF might function via presynaptic tropomyosin receptor kinase B (TrkB) to ensure fast, reliable neurotransmitter release in the developing brain [52]. Moreover, this mechanism was recently described to play a role in cognitive impairment [54]. Another promising molecule is GS. The study illustrated that depletion of OL GS disrupts neuronal glutamate signaling with accompanying changes in behavioral function [53]. Interestingly, OLs survival and myelination were unaffected, thus demonstrating a myelin-independent role for oligodendrocytes in supporting glutamate signaling in the brain [53].

#### GABA

Among chemokines, CXCL12 is the most widely studied and was demonstrated to exert diverse electrophysiological effects in distinct neuronal populations within the same or different structures. Specifically, by CXCR4 activation in the DRN, CXCL12 enhanced sIPSC frequency on serotonergic neurons, whereas it reduced sIPSC amplitude in non-serotonergic neurons [81], suggesting postsynaptic and presynaptic mechanisms, respectively. Evidence from another electrophysiological study has illustrated that CXCL12 increases the release of GABA from hypothalamic melanin-concentrating hormone (MCH) neurons, which affects the excitability of these cells in a dose-dependent manner [80]. Similarly, in the SN, CXCL12 enhanced the frequency of sIPSCs and mIPSCs indicating presynaptic mechanisms, consistent with the documented presence of CXCR4 on the GABAergic neurons [17]. Furthermore, following CXCL12 treatment, Purkinje cells in the cerebellum showed an increase in the spontaneous GABAergic activity by CXCR4 activation [99]. Interestingly, CXCR4 is associated with outward G protein-coupled inward rectifying potassium, GIRK, channel. This current was shown to be prevented by applying the GABA_B_ antagonist, suggesting GABA spillover onto GABA_B_ receptors [100] as well as CXCR4-exerted actions by additional GABAergic receptors (see Table 2).

Regarding CX3CL1 actions on GABAergic transmission, this molecule was shown to increase the amplitude of sIPSC and eIPSC without affecting the paired-pulse ratio (PPR). These effects lead to an increased sensitivity of serotoninergic DRN neurons to GABA inputs, thereby indirectly inhibiting serotoninergic neurotransmission [101].

Although CXCL16 and CCL2 upregulated excitatory synaptic transmission in the hippocampus, they did not affect inhibitory synaptic transmission in this area [90]. However, CCL2 was reported to reduce GABAergic transmission in the spinal cord and thus increase spinal neuronal excitability leading to disinhibition [102].

In support to astrocytic regulation of synaptic transmission, evidence illustrated that based on frequency and duration of interneuron firing, astrocytes could tune in to release either glutamate, which enhances excitatory synaptic activity [103], or ATP/adenosine, which decreases excitatory synaptic strength [104].

#### Dopamine

CCL2 and CXCL12, together with their corresponding receptors, CCR2 and CXCR4, are constitutively expressed in the SN’s dopaminergic neurons (DA, see section “Cellular distribution of chemokine and chemokine receptors in the brain”). Moreover, despite differences in channel modulation between CCL2/CCR2 (a closure of potassium channels) and CXCL12/CXC4 pairs (an increase in the N-type channel, one of high voltage-activated channels), they both produce a similar effect: the increase in excitability of dopaminergic neurons of SN, which leads to an enhanced DA release ([105, 106], see Table 2). This notion was further supported by in vivo study, where intranigral CCL2 application resulted in an ipsilateral increase in dopamine and its metabolites and further behavioral outcome, as described in section 2.4 [107].

These data strongly suggest that the CXCL12 can act directly as a neuromodulator of dopaminergic neuronal electrical activity by affecting HVA currents. Moreover, in the same neuronal population, CXCL12 application leads to an increase in an inward current resistant to tetrodotoxin (TTX). It was speculated that its source might be glutamate secreted from non-neural cells [17]. As CXCR4 was not present on astrocytic cells in the SN [108], thus this inward current likely resulted from the activation of CXCR4 on microglia. However, further experiments need to verify this hypothesis.

#### Neuropeptides

In addition to DA neurons, CCL2 and CXCL12 were reported to co-localize with neurons releasing neuropeptides, such as vasopressin (AVP) and melanin-concentrating hormone (MHC) in posterior pituitary neurons, and substance P in DRG neurons (see “Cellular distribution of chemokine and chemokine receptors in the brain”). These findings suggest that CCL2 and CXCL12 cognate receptors, CCR2 and CXCL12, are involved in the autocrine regulation of these neurons. Consistent with this idea, CXCR4 was described to directly modulate several voltage-dependent channels, including Na^+^ and K^+^ channels of the action potential in MCH neurons [80] or tetrodotoxin-sensitive sodium currents in hypothalamic neurons [109]. Moreover, both of these chemokines are packed in small dense vesicles in the nerve terminals of these neurons, which are then released from neuronal cell bodies and terminal nerve endings in a calcium-dependent manner (for example: [110]).

Furthermore, electrophysiological recordings of supraoptic neurons have demonstrated that CXCL12 decreases the electrical activity of AVP neurons via CXCR4, resulting in modifications in AVP release. In addition, intracerebroventricular injections of CXCL12 were illustrated to significantly inhibit AVP release induced by high plasma osmolarity [111].

Recent findings cast a new light on the role of astrocytic gap junction networks in neuroendocrine functions [112]. In particular, mice with deletion of Cx43 in astrocytes displayed much less excitable orexin neurons located in the lateral hypothalamic area (LHA) due to dysfunction in glucose and lactate trafficking, leading to behavioral dysfunctions manifested as impaired sleep–wake cycle [112]. Thus, again, keeping in mind ACKR3-induced Cx43 internalization, it is intriguing to hypothesize that activation of this receptor could exert similar actions. However, this hypothesis needs further investigation.

#### Serotonin

As described above, both CXCL12 and CX3CL1 were found to modulate GABAergic synaptic transmission onto serotoninergic neurons. CXCL12 was shown to increase sIPSC frequency, which may inhibit the excitability of serotoninergic cells and the release of its neurotransmitter. In contrast, CX3CL1 increases the amplitude of sIPSCs and eIPSC without affecting the PPR, increasing the sensitivity of serotoninergic neurons to GABA inputs and consequently inhibiting 5-HT neurotransmission [113]. Similarly, CCL2 induced hyperpolarization and a reduction in the firing of 5-HT neurons of the DRN [114].

Thus, despite different mechanisms of modulation of GABAergic transmission, the electrophysiological output is similar, and to date, all chemokines are suggested to inhibit serotoninergic cells in DRN.

#### Opioids

Convincing evidence illustrates that opioid receptors and CKRs are often co-expressed in the same neuron and can directly interact, thus each influencing the function of the other (see “CXCL12/CXCR4/ACKR3”). In this regard, the most studied mechanism is the heterologous desensitization, where the activation of one type of GPCR receptor leads to the inactivation of another GPCR receptor present in the same cell. Consistent with this idea, following CXCL12 exposure, the heterologous desensitization of opioid receptors has been linked to functional outcomes at the cellular (e.g., reduced chemotaxis [115]) and electrophysiological (e.g., decreased morphine-induced hyperpolarization in PAG neurons in the presence of CXCL12 [113]) levels.

A recent elegant study has provided extensive evidence that ACKR3 is a chemokine receptor with the ability to bind opioid peptides; however, opioid binding did not trigger downstream signaling through this receptor [21]. Thus, it is suggested that ACKR3 serves scavenger functions for many opioids, especially enkephalins and dynorphins, by reducing their availability for their classical opioid receptors [21]. Accordingly, treatment with ACKR3 agonist LIH383, even at high concentration, did not produce any electrophysiological effect in PAG neurons, confirming the scavenging function of ACKR3 in this brain region. Interestingly, in the presence of the ACKR3 agonist, dynorphin A improved its potency towards its classical receptors, which resulted in more significant inhibition of neuronal firing than in the absence of ACKR3 activation [21]. These findings suggest that ACKR3 regulates opioid peptide availability, and consequently, their signaling through opioid classical receptors [21].

#### Dendritic spines

Synaptic transmission is explicitly related to dendritic spines, as these tiny, actin-rich, postsynaptic protrusions from a dendrite receive most of the excitatory synaptic inputs in the brain [116]. Functional and structural changes in dendritic spines are also critical for synaptic plasticity (see “Chemokine action at the network level”), a cellular model of learning and memory. Moreover, the spine morphology is highly dynamic: it could vary within the same dendritic arbor and thus is interdependently regulated.

In agreement with glial contribution to synaptic functions, astrocytes [117], microglia [118], and oligodendrocytes [119] are implicated in interactions with dendritic spines in a highly active-dependent manner. The first evidence of a link between chemokines and dendritic spines was provided by Paolicelli et al. [118]. Deficient of CX3CR1 microglia displayed impairment in synaptic pruning, reflected as a transient deficit in excitatory synaptic transmission and a reduced synaptic density as compared to their wild-type littermates, indicating immature synaptic properties. These changes were present during the early postnatal stage (P8–P28) and then returned to baseline, highlighting CX3CR1 impact on synaptic pruning during neurodevelopment. However, these mice were also tested in similar experimental settings in their adulthood (P40) [120]. Interestingly, despite unchanged dendritic spine density, neurons from CX3CR1^−/−^ mice displayed a lower frequency of mEPSCs and sEPSCs compared to their wild-type littermates. These results illustrate that a transient reduction in microglial synaptic pruning during development is sufficient to induce the long-term deficit in synaptic multiplicity, whereas the number of excitatory synapses remained the same [120]. These data support the idea of a critical window during which microglia-mediated synaptic pruning is crucial for proper circuit maturation.

Similarly, CX3CL1 impact on dendritic spines was documented investigating newborn cells. Using the same strain of mice, it was documented that adult transgenic animals displayed a reduced number of dendritic spines with morphological changes, reflected as enlargement and shortening, in adult-born granule neurons in the dentate gyrus (DG) [121]. Despite being ultrastructurally enlarged, synapses at both afferent and efferent levels were depleted of synaptic vesicles, suggesting impaired functionality.

Moreover, dysfunctions in glutamatergic neurotransmission in CX3CR1 KO mice were further confirmed in CA3–CA1 synapses in hippocampal slices [122]. Recordings from slices of transgenic mice displayed an immature AMPA/NMDA ratio with defective AMPA components and decreased release probability compared to wild-type mice. These results illustrate that neuron–microglia interactions profoundly influence the functional maturation of excitatory presynaptic function.

CXCL12 is another chemokine that is documented to be involved in dendritic spine modifications [124]. Specifically, this chemokine increases dendritic spine density in cerebral cortex human neurons. For example, CXCR4 activation by CXCL12 was reported to positively regulate neuronal survival and dendritic spine density in cortical neurons. However, the molecular mechanisms underlying CXCR4 regulation of dendritic spines are not completely defined [125].

Specifically, CCR5 is involved in the modulation of dendritic spine turnover and clustered spine addition in the mouse retrosplenial cortex, both of which correlate with learning and memory performance [126].

Taken together, as expected from their complex expression of cellular pattern and dynamic chemokine–receptor interactions, chemokines modulate intrinsic membrane properties, synaptic transmission, and gliotransmission by implementing diverse cell- and region-specific mechanisms. However, noting the extensive evidence of chemokine actions on excitatory synaptic transmission and synaptic plasticity, it is tempting to speculate that chemokine actions on dendritic spines are still largely unexplored.

### Chemokine action at the network level

The finding that chemokines exert a neuromodulatory role in the brain is essential for a better understanding of the complex communication network between neurons and cells from their surrounding microenvironment. This section illustrates the emergent concept of chemokine coordination of neural networks, such as neuroendocrine functions, GABA receptor-mediated synaptic transmission, and synchronized oscillatory activity.

#### Interneurons

All neuronal circuits of higher animals comprise excitatory and inhibitory neurons forming intense feed-forward and feedback connections [129]. However, the precise control of network operations would not be possible without interneurons. Although they constitute only 20–30% of all cells in cortical circuits, this small, diverse group of inhibitory neurons enables higher brain functions, such as working memory, cognitive flexibility, attention, or social interaction (for a review: [130]). Through inhibition, GABAergic interneurons can shape the circuit activity controlling (1) frequency of surrounding pyramidal neurons, (2) network excitation level by feed-forward mechanism, and (3) synchronized oscillation events [130].

The importance of interneurons in relation to network functioning illustrates the highly controlled manner by which interneurons connect with other neurons [131]. The best-characterized feature of constitutively expressed chemokines in the brain, particularly CXCL12 and CX3CL1, is the regulation of cell and axonal/neurite migration during neurodevelopment. Noting their documented modulation of GABAergic transmission (see “GABA”) may indicate that chemokines participate in this process. Consistently, CXCL12/CXCR4 axis was demonstrated to guide interneurons (for example: [132]) and promote the maturation of GABAergic neurons during neurodevelopment [133]. Moreover, as expected, perturbation of CXCL12/CXCR4 signaling, e.g., in their gradients, leads to premature cortical plate invasion by cortical interneurons and, in the long term, disturbance in their laminar and regional distribution [134], leading to the dysfunctional neural network.

Disturbances were also present regarding CX3CL1/CX3CR1 pathway, which plays an essential role in neurodevelopment. Similar to CXCL12, the CX3CL1/CX3CR1 axis was demonstrated to affect the outgrowth of dopaminergic axons in the forebrain and the laminar positioning of subsets of cortical interneurons [135]. Moreover, CX3CR1 deficiency impairs the functional maturation of thalamocortical synapses in the developing barrel cortex, indicating crucial involvement of CX3CL1/CX3CR1 signaling in this process [136]. As the CX3CR1 receptor is mainly expressed by microglia, and those cells display a well-documented process of network maturation, called synaptic pruning, microglia are likely to participate in the maturation of synapses. Therefore, these findings highlight the importance of chemokine regulation of proper neural network functioning by their effects on interneurons migration and maturation.

Neuronal growth and synapse formation can also be achieved by a phenomenon called giant depolarizing potentials (GDPs). GDPs are spontaneous network-driven rhythmic-like large neuronal oscillations in the developing hippocampus, formed by GABA, exerting depolarizing and excitatory actions during postnatal development. CXCL12 was reported to tonically inhibit GDPs, further highlighting the role of this chemokine in neuronal development [137]. Moreover, they are modulated by external factors, including chemokines, such as CXCL12, CX3CL1, and CCL2 [2, 67, 138].

Importantly, Sheridan et al. [67] reported that the CX3CL1 application in the hippocampus has opposing effects on glutamate-induced Ca^2+^ transients depending on whether it was applied alone or simultaneously with a GABA_A_ blocker, picrotoxin. These results highlight the possible modulation of CX3CL1 in regulating the balance between excitation and inhibition on the network level. However, whether the chemokine system participates in feed-forward inhibition signaling remains an open question for future research.

#### Synaptic plasticity

Synaptic plasticity is defined as a molecular form of learning, memory, and development in neural circuits. It mainly comprises such mechanisms as Hebbian plasticity, synaptic scaling, and adult neurogenesis. Among different factors regulating synaptic plasticity, glial cells have been considered key players in maintaining synapse homeostasis [139–142].

##### Hebbian plasticity—LTP and LTD

Hebbian synaptic plasticity is the earliest and most widely accepted neurobiological theory of memory consolidation. According to this concept, a synapse will be strengthened if the presynaptic neuron is active while the postsynaptic neuron is firing, a process called Hebbian modification.

As mentioned earlier in the section on synaptic transmission (see “Glutamate”), in the CA1 hippocampus, CX3CL1 induced phosphorylation of AMPA subunit GluR1 at postsynaptic sites [82], which is one of the hallmarks of the LTD. Moreover, one group reported no difference in LTD in the hippocampus between adult wild-type and knockout mice. In contrast, LTD is enhanced in CX3CR1^−/−^ mice at P13 [118], suggesting that CX3CL1 has the highest relevance during early postnatal age.

However, similar electrophysiological studies indicated either a complete lack of LTP in knockout mice [143] or a more pronounced LTP than in wild-type counterparts when LTP was induced by a weak stimulation protocol [144]. Interestingly, when mice were housed in an enriched environment compared to wild-type mice, they had enhanced plasticity [145]. These results suggest that the absence of microglia-driven CX3CR1 signaling impedes enrichment effects, possibly due to some shared mechanisms that warrant further investigation. It is speculative that this lack of beneficial response to environmental stimulation might be explained by altered microglia–neurons cross-talk, affecting the circuitry underlying synaptic plasticity.

Regarding the DG, CX3CL1 decreased the LTP at those synapses; however, when GABA_A_ receptors were blocked, the magnitude of the LTP was enhanced [67], indicating that the CX3CL1-exerted actions may differ based on inhibitory/excitatory balance (see Table 2). Alternatively, noting the CX3CL1-induced potentiation of NMDA function and thereby the increase in calcium transients [72] together with the inhibition of the LTP induction following NMDAR activation, it is also possible that CX3CL1-induced elevated level of intracellular calcium could interfere with the LTP induction [72].

These inconsistent findings between laboratories might result from differences in age, gender, diet, housing conditions, or electrophysiological protocols. Especially age and gender could be relevant, as the same laboratories cannot replicate their results when those variables had differed. Hence, effects on synaptic plasticity and behavioral outcomes resulted from the CX3CL1–CX3CR1 axis may be transient and quickly compensated in knockout mice.

Chemokine regulation of synaptic plasticity is not restricted to the hippocampus. CX3CL1 was shown to induce synaptic depression of evoked parallel fibers inputs onto Purkinje neurons [146], and mice with CX3CR1^−/−^ receptor deletion were characterized by the LTP in the spinal cord [147]. Future research should clarify whether mechanisms underlying CX3CL1-induced synaptic plasticity are general.

However, it would be implausible, considering that (1) microglia displays many region-specific features, especially when compared in cerebellum and cortex [148], (2) CX3CR1-induced microglial during development are region-specific [149], as well as (3) mechanisms governing LTP may differ between structures, for instance, between the hippocampus and spinal cord [150].

In addition, another chemokine, CCL3, one of the CCR5 ligands (see “CCL3/CCL4/CCL5/CCR5”), was also shown to significantly reduced basal synaptic transmission and LTP at the Schaffer collateral-CA1 hippocampal synapses [87]. Furthermore, CCL3-induced LTP impairment was completely prevented by co-injection with maraviroc, a blocker of the CCR5 receptor.

##### Synaptic scaling

To prevent the destabilizing component of Hebbian plasticity in network function, neurons are able to sense their excitability and trigger negative-feedback homeostatic mechanisms to counteract perturbations in synaptic activity and restrain it within a dynamical but physiological range [140]. Recent accumulating data also highlight the essential role of glial cells, as they are equipped with, for instance, glutamate transporters to control the concentration of glutamate and other neurotransmitters in the microenvironment. Importantly, stable but flexible neural activity is fundamental for proper brain function and support of animal behavior, depending on a dynamic interface between homeostatic synaptic plasticity mechanisms and Hebbian forms of plasticity [131, 140].

It is widely accepted that TNFα mediates synaptic scaling, a form of homeostatic plasticity leading to the adjustment of synaptic strength [151]. Glia, mainly astrocytes, have been identified as the main source of TNFα in the CNS. Therefore, by modulating TNFα levels, glia participate in homeostatic activity-dependent regulation of synaptic plasticity [151]. As it is well documented that CXCR4 is mainly expressed on astrocytes, CXCL12/CXCR4 pathway is also involved in regulating synaptic plasticity. Moreover, evidence also implicated microglia in the regulation of synaptic plasticity, as they release TNFα [152].

##### Adult neurogenesis

Adult neurogenesis in the rodent brain is thought to reside in two niches, the SVZ adjacent to the lateral ventricles and the subgranular zone (SGZ) located in the DG. Division, maturation, and forming functional synapses or apoptosis are key processes that need to be tightly controlled to maintain an adequate balance between plasticity and stability. As expected, more and more data shows an increasing impact of glial cells and chemokines on this phenomenon. Consistently, increasing studies implicated both exogenous or exercise-induced CX3CL1 in promoting neurogenesis and NSC/NPC activity in a microglia-dependent manner, whereas depletion of CX3CR1 resulted in a decreased number of newly generated neurons [143]. Moreover, morphological alternations in mature granule cells were still present in CX3CR1 deficient mice [121], suggesting their long-lasting changes. Similar to CX3CL1, CXCR5 was also reported to enhance the proliferation of SGZ cells in the DG. Nonetheless, no difference in microglial density or their activation was observed [155], implying microglia-independent action.

#### Neuroendocrine circuit

The hypothalamus, a brain region comprised of different nuclei and several neuronal populations producing neuropeptides, is critically involved in the regulation of major neuroendocrine functions, including water/food intake, metabolism, and also stress.

Depending on the concentration, CXCL12 is thought to exert opposite effects on the action potential discharge [80], which may have functional consequences on the activity of neuroendocrine networks. Specifically, CXCR4 is tonically active at low physiological concentrations, constantly exerting its action on MCH neurons. Under basal conditions, those cells showed more hyperpolarized membrane potential than orexin neurons, which do not express CXCR4 [156]. Moreover, CXCL12/CXCR4/ACKR3 signaling dysfunctions are associated with feeding disorders, such as anorexia. Consistently, a high-fat diet was reported to increase the expression of CXCL12, its receptors, and neuropeptides, such as enkephalin, galanin, orexin, and MCH in the PVN and perifornical lateral hypothalamus [157]. Importantly, intracerebroventricular injection of CXCL12 was shown to mimic those effects of the high-fat diet in the hypothalamus, suggesting a vital role of the CXCL12/CXCR4/ACKR3 axis in feeding behavior [157].

#### Functional connectivity

Considering the relevance of chemokines’ role in neurodevelopment (i.e., neuronal/axonal migration and synaptic pruning), it can be hypothesized that proper chemokine signaling during neurodevelopment is of high importance to provide functional connectivity between many cortical regions. However, to date, there is a scarcity of data on this topic. In particular, by measuring connections between hippocampus and prefrontal cortex, mice with deletion of CX3CR1 revealed a decrease in the coherence between those two structures, compared to their wild-type littermates ([120], see Fig. 4D). Thus, this finding suggests that the depletion of CX3CR1 leads to a decrease in hippocampal–prefrontal connectivity, which may have behavioral consequences, such as anxiety or cognitive impairments [120].

Moreover, knockout mice showed reduced frontal cortex oscillatory driving to the hippocampus than their wild-type littermates at baseline conditions [158]. It is speculative whether it is the consequence of morphological modifications of microglia depleted of CX3CR1 [51], but scanty evidence is available so far. Nevertheless, these results highlight a fundamental role of CX3CL1 at the network level. More recently, in another study, the expression of one chemokine, CXCL1, was elevated in the visual cortex, which was exposed to gamma frequency synchronization [159]. Hence, considering those findings and accumulating evidence of glial impact on the network, investigating chemokines’ role in neural networks may be an exciting direction in the future.

### Chemokine action on behavior

Since mounting evidence supports the notion that chemokines coordinate several brain circuits of behavioral relevance, including the hippocampus, DRN, SN, hypothalamus, disturbances of several chemokines/chemokine receptor axes were reported in the pathophysiology of neurobiological disorders [157, 160, 161]. Here we discuss a handful of studies on the regulation of chemokines on various types of behavior, such as learning and memory, social interactions, locomotor activity, anxiety. Results from those studies are summarized in Table 3.

**Table 3:** Summary of chemokines action in different behavioral tests

Receptor/chemokine	Reference	Strain	Age (months)	Sex	Test/paradigm	Results
Anhedonia						
CX3CR1	[181]	CX3CR1^KO/KO^	2–3	M	Sucrose consumption test	Resilience after chronic stress
CX3CR1	[171]	CX3CR^KO/KO^	3	M	Saccharin preference test, chronic unpredictable stress	Resilience after chronic stress
CX3CR1	[184]	CX3CR1^KO/KO^	3–4	M	Sucrose preference	ND
Anxiety/locomotor activity						
CX3CR1	[143]	CX3CR1^KO/KO^	3	M	EPM, OF	ND
CX3CR1	[166]	CX3CR1^KO/KO^	2–3	both	EPM, OF, wire hang test	ND
CX3CR1	[167]	CX3CR1^KO/KO^	2	M	Acoustic startle	ND
CX3CR1	[167]	CX3CR1^KO/KO^	2	M	EZM, LDB, OF	↓ Anxiety-like behavior
CX3CR1	[181]	CX3CR1^KO/KO^	2–3	M	OF, EPM	ND, anxiety-like behavior after chronic stress (only OF)
CX3CR1	[121]	CX3CR1^KO/KO^	1.5	F	EPM, OF	The hyperactive and anxiolytic-like phenotype in KO mice
ACKR3	[20]	C57BL/6 J	6	F	EPM	↑ Both entries into open arm and time spent there when ACKR3 blocked
ACKR1	[176]	ACKR1^−/−^	6	M	EPM	↑ Anxiety
Learning and/or memory						
CX3CR1	[143]	CX3CR1^KO/KO^	3	M	Rotarod test, fear-conditioning, water Morris maze	↓ Motor learning (but not motor coordination) in KO, ↓ Contextual learning, and memory (dependent on IL-1β), ND in fear-conditional learning
CX3CR1	[144]	CX3CR1^KO/KO^	1	F	Water Morris Maze	↑ Learning, not affected by environmental enrichment
CX3CR1	[120]	CX3CR1^KO/KO^	0.5–1	both	Maternal homing test, NOR	ND—an intact ability to detect and respond to familiar olfactory cues and similar capacity and motivation to explore inanimate objects in KO
CX3CR1	[120]	CX3CR1^KO/KO^	2–4	M	Social preference test	ND in preference for either tube, demonstrating deficient social interaction in CX3CR1^−/−^ mice
CX3CR1	[167]	CX3CR1^KO/KO^	2	M	Contextual fear conditioning	↑ Fear acquisition and ↑ fear reinstatement in CX3CR1^−/−^ mice
CXCR4	[170]	Wistar, icv. injection of CXCR4 antagonist	1.5–2	M	NOR	↓ Memory
CCR5	[162]	CCR5^KO/KO^	3	M	contextual fear-Conditioning, water Morris Maze	↑ Learning and memory, including spatial memory
CCL2	[87]	C57BL/6 J, icv. injections of CCL2	2- 3	M	Two-step Y-maze; passive avoidance tasks	↓ Short term memory and learning
CCL2	[182]	CCR5^−/−^ mice on the DBA1/J background	1	M	Water Morris Maze; fear-conditioning	ND
ACKR1	[176]	ACKR1^−/−^	6	M	Water Morris Maze	↓ Memory
CX3CR1	[184]	CX3CR1^KO/KO^	3–4	M	NOR	ND
ACKR1	[176]	ACKR1^−/−^	6	M	Water Morris Maze	ND in acquisition learning and visual acuity
Depressive-like						
CX3CR1	[166]	CX3CR1^KO/KO^	2–3	Both	TST, FST	Resilience after chronic stress
CX3CR1	[181]	CX3CR1^KO/KO^	2–3	M	FST, TST	↑ Coping behavior in KO mice
CX3CR1	[121]	CX3CR1^KO/KO^	1.5	F	TST	↑ Depression-like behavior
Motor activity						
CXCR4	[107]	Wistar, intranigral injection of CXCL12	1	M	Circling behavioral test	↑ The number of contralateral turns, but not ipsilateral after unilateral intranigral injection
CCL2	[173]	Wistar, icv. injection of CXCL12	2.5–3	M	Actimeter	↓ In the motor activity
CCL2	[106]	Wistar, intranigral injection of CXCL12	2.5	M	Circling behavioral test	↑ The number of contralateral turns, but not ipsilateral after unilateral intranigral injection
ACKR1	[176]	ACKR1^−/−^	6	M	Actimeter, rotarod test	↓ Locomotion and imbalance in KO
Social activity						
CCR5	[162]	CCR5^KO/KO^	3	M	Social recognition task	ND
CCR5	[162]	CCR5^KO/KO^	3	M	EPM, OF	ND
CCL2	[182]	CCR5^KO/KO^ mice on the DBA1/J background	1 month	M	Social test recognition	↑ Social recognition in KO mice, ↓ social recognition after CCL2 injection
CX3CR1	[51]	CX3CR1^KO/KO^	1.5–2	M	Social dominance test	Social submissiveness of CX3CR1^G/G^ mice compared to both CX3CR1^+/+^ and CX3CR1^+/G^ mice

*ND* no differences, *EPM* elevated plus maze, *EZM* elevated zero maze, *OF* open field, *LDB* light–dark box, *NOR* novel object recognition, *TST* tail suspension test

#### Learning and memory

As described in the section on synaptic plasticity (see “Hebbian plasticity—LTP and LTD”), several chemokines were documented to modulate LTP in the hippocampus, a cellular mechanism underlying learning and memory [88, 121, 143, 162]. Of these chemokines, CX3CL1 is the best characterized in this context. Behavioral studies on the CX3CL1/CX3CR1 axis in the regulation of learning and memory were giving contradictory results (see Table 3). Briefly, CX3CR^−/−^ mice have shown impaired [143], improved [144, 163] or unaltered [164] cognitive functions. It should be noted that findings from studies of synaptic plasticity correlate with behavioral output (see Tables 2, 3). Those discrepancies may result from differences in housing conditions, animal gender, age, or behavioral test protocols between laboratories. However, this issue warrants further clarifications.

Increasing evidence has indicated the role of another chemokine receptor, CCR5, in cognitive functions (Table 3). While the activation or overexpression of CCR5 was reported to impair the hippocampal and cortical neuronal plasticity, leading to deficits in learning and memory [162], both genetic depletion and pharmacological inhibition of CCR5 was shown to enhance memory in rats [165], as measured by both fear-conditioning (contextual learning) and Morris water maze (spatial learning, see Table 3).

Hence, these findings indicate that implications to memory-related functions are not specific for CX3CR1 action, and studies have just begun to elucidate the mechanisms and other targets implicated in memory and learning.

#### Locomotor activity

Since many chemokines with their cognate receptors are expressed in dopaminergic neurons and regulate their activity to induce the release of DA (see Sections “Dopamine” and “Cellular distribution of chemokine and chemokine receptors in the brain”), the behavioral relevance of their actions was further investigated. Using unilateral intranigral or intracerebroventricular injections of CXCL12 or CCL2 in the rat SN, it was shown that both of these chemokines induced contralateral turns in circling behavior [106, 107]. These data are consistent with CXCL12- or CCL2-induced dopamine release from dopaminergic neurons in substantia nigra (see “Dopamine”). It is worth noting that when chemokines were injected through the SN, animals’ locomotor activity increased, resulting in the contralateral turning [106, 107]. However, when CCL2 was injected intracerebroventricular, the animal locomotor activity was decreased.

Moreover, a recent study revealed the involvement of microglia and their receptor CX3CR1 in the adaptive response to psychosocial stress, which is a major risk factor for psychotic disorders such as schizophrenia [51].

#### Emotional behavior

Besides its role in memory and learning, CX3CR1^−/−^ knockout mice have attracted attention regarding chronic stress. Briefly, CX3CR1 was indicated to control social behavior and social dominance ([51, 120], Table3). However, there are some inconsistencies. Considering anxiety, most studies did not show any differences between genotypes [167]. Nonetheless, there is one study that suggests anxiolytic effects of CX3CR1 depletion [121], whereas the other study indicates the opposite ([167], Table3). A similar situation was described regarding depressive-like behavior: one study indicates CX3CR1^−/−^ knockout is more depressive measured by Forced Swim Test and/or Tail Suspension Test, whereas the other reported the opposite conclusion. It seems like the main difference between those experiments was the gender between animals. Specifically, CX3CR1^−/−^ females are more depressive and anxiolytic than wild-type littermates, whereas the depletion of the same receptor in males elicits the opposite reaction. However, further investigation is needed.

Consistent with a recent report on ACKR3 and opioids crosstalk (see “Opioids”), along with implications that the modulation of circadian glucocorticoid oscillation may affect emotional behavior (e.g. [168]), it was shown that ACKR3 binds intermediate opioid peptides, specifically BAM22 and peptide E, which results in enhancement of circadian glucocorticoid oscillation in the adrenal glands, and, therefore, anxiolytic-like behavior [20]. BAM22 and peptide E were shown to be released under a condition named subcapsular cell hyperplasia (SCH) [20], which is a pathohistological change of adrenal tissue. SCH cells lack prohormone convertase, an enzyme required for processing these proteins to mature enkephalins [20]. Therefore, this study highlights the functional relevance of chemokine and opioid systems interactions.

To sum up, these findings reinforce the idea that chemokines and their receptors are involved in higher brain functions regulating many types of behavior, including cognitive, motor, and emotional effects. However, to date, very little is known about their cellular, molecular, electrophysiological, and glial-mediated mechanisms to put these results in a broader context.

## Concluding remarks

A great line of evidence suggests that chemokines are a unique class of neuromodulators, which regulate many biological aspects as diverse as neurodevelopment, neuroinflammation, and synaptic transmission. As for a long time chemokines were investigated and thought of only in the context of inflammation, there are still many crucial aspects to elucidate under physiological conditions. For instance, despite their well-known ability to modulate intrinsic membrane properties and inhibitory transmission in the brain, as well as chemokines’ impact on memory, to our best knowledge, there is no data on their role in neuronal oscillations. Moreover, we believe that the dynamical and interdependent mechanisms of chemokines–receptor interactions together with chemokines’ coordination of intercommunication between neurons, glia, and endothelial cells generate a complex regulatory network that allows the chemokine system to fine-tuning many brain functions. The real challenge is in studying such interdependent and interconnected mechanisms and planning an experimental design, which requires a high level of control of major variation. As further elements of these complex interactions are identified, new opportunities for drug discovery efforts targeting specific functional outcomes of the receptor will likely emerge.

## Abbreviations

A_1_R: Adenosine A_1_ receptor
A_2A_R: Adenosine A_2A_ receptor
A_3_R: Adenosine A_3_ receptor
AVP: Vasopressin
CKR: Chemokine receptor
CNS: Central nervous system
DA: Dopamine
DG: Dentate gyrus
DRG: Dorsal horn of the spinal cord
DRN: Dorsal raphe nucleus
eEPSCs: Evoked excitatory postsynaptic current
eIPSC: Evoked inhibitory postsynaptic current
GAGs: Glycosaminoglycans
GDPs: Giant depolarizing potentials
GFP: Green fluorescent protein
GPCRs: G-protein-coupled receptors
GS: Glutamine synthetase
LTD: Long-term depression
LTP: Long-term potentiation
MCH: Melanin-concentrating hormone
mEPSCs: Evoked excitatory postsynaptic current
MS: Multiple sclerosis
OLs: Oligodendrocytes
OPCs: Oligodendrocyte progenitor cells
PPR: The paired-pulse ratio
PTMs: Post-translational modifications
sEPSCs: Spontaneous excitatory postsynaptic current
SGZ: Subgranular zone
sIPSCs: Spontaneous inhibitory postsynaptic current
SN: Substantia nigra
SVZ: Subventricular zone
TNFα: Tumor necrosis factor α
TTX: Tetrodotoxin
